# Comparison of two UPS regulators: the 26S proteasome LID and the COP9 signalosome

**DOI:** 10.3389/fcell.2024.1496862

**Published:** 2024-11-12

**Authors:** Dawadschargal Dubiel, Wolfgang Dubiel

**Affiliations:** Institute of Experimental Internal Medicine, Medical Faculty, Otto von Guericke University, Magdeburg, Germany

**Keywords:** 26S proteasome LID, COP9 signalosome, cullin–RING ubiquitin ligase, metallodeubiquitylase, PCI domain proteins, MPN domain proteins, MPN^+^ domain proteins

## Introduction

In this work, we would like to compare two essential regulators of the ubiquitin proteasome system: the 26S proteasome (Hough et al., 1987) LID (Glickman et al., 1998) and the COP9 signalosome (CSN) (Chamovitz et al., 1996; Seeger et al., 1998). The LID and the BASE form the 19S regulator (RP), which determines the 20S proteasome or core particle (CP) action (Gorbea et al., 1999; Finley and Prado, 2020). Although the LID has a metallodeubiquitylase activity on the subunit RPN11 to cleave off the ubiquitin labeling of substrates, the BASE is formed by six heteromeric ATPases, which denature and linearize substrates in an ATP-dependent fashion before they enter the CP for degradation (Finley, 2009). The LID is a paralog particle of the CSN, which forms permanent complexes with cullin–RING ubiquitin ligases (CRLs), regulating the E3 ligases (Dubiel et al., 2023). The paralog subunit of RPN11 in the CSN, CSN5, has a similar metallodeubiquitylation active site, which removes NEDD8 from cullins inactivating CRLs (Cope et al., 2002). Active CSN-CRL complexes produce ubiquitin conjugates which are deubiquitylated and denatured in the RP for transfer into the PC for degradation.

Many of the PC regulators were discovered in Rechsteiner’s laboratory. The RP (Rechsteiner et al., 1993) consisted of the BASE subunits S4 or RPT2 and S7 or MSS1 or RPT1 (Dubiel et al., 1992a; Dubiel et al., 1993) and the LID subunit (Gorbea et al., 1999) S12 or Mov34 or RPN8 (Dubiel et al., 1995) and others. A completely different 20S modifier is the 11S regulator (Dubiel et al., 1992b) or PA28 (Ma et al., 1992), which is engaged in antigen processing/presentation (Rechsteiner et al., 2000; Schwarz et al., 2000). A nuclear CP activator, called proteasome activator 200 (PA200), was characterized in *Mammals*, forming a complex with the 20S proteasome (Ustrell et al., 2002). Furthermore, *Mammalian* ECM29, a 26S proteasome-associated protein, was identified determining the localization of the 26S proteasome (Ustrell et al., 2002). We were interested on whether the CSN is a regulator of the 20S enzyme.

## The 26S proteasome LID and the COP9 signalosome: two paralog complexes

**FIGURE 1:**
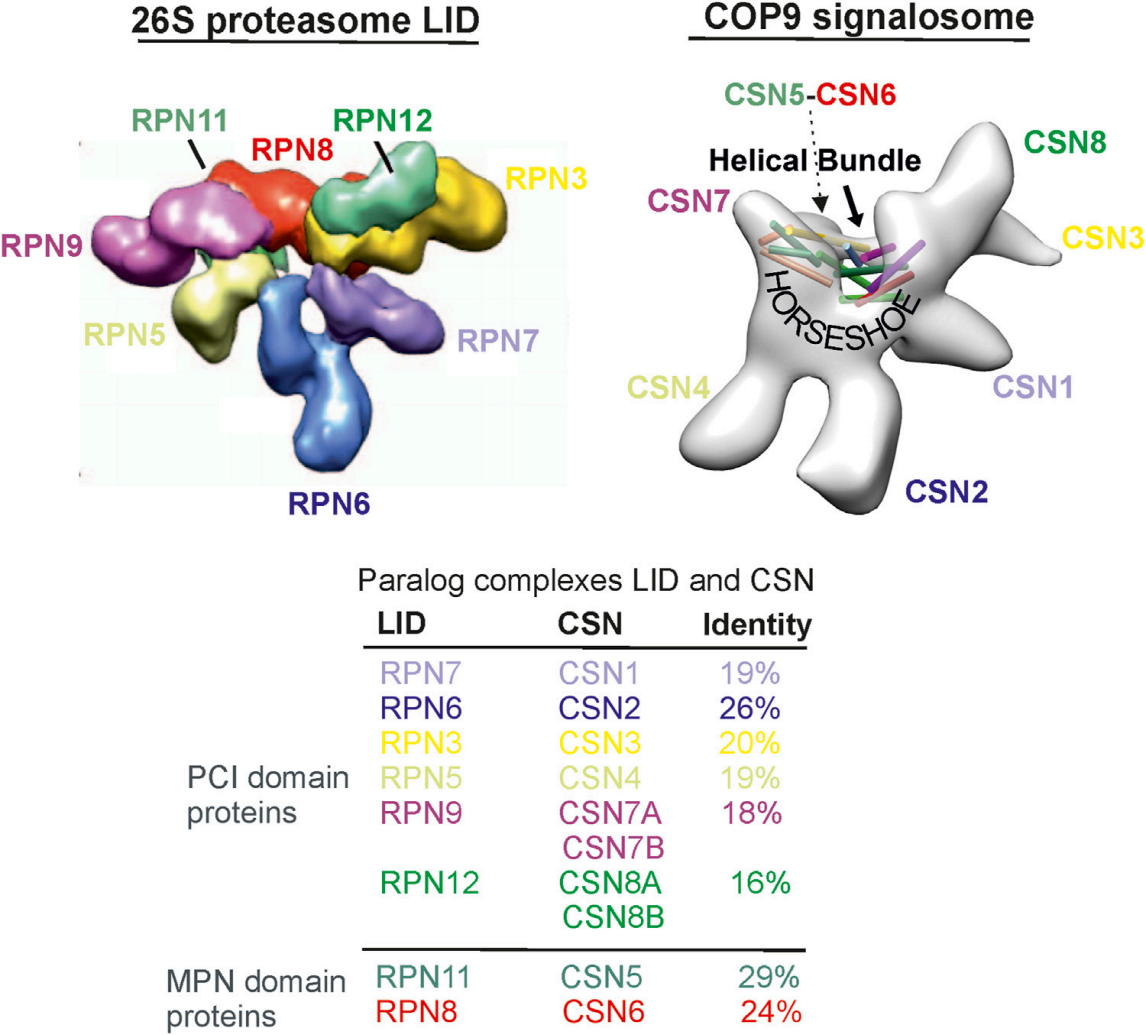
The 26S proteasome LID particle structure is taken from Martin and co-workers (Lander et al., 2012). The CSN particle originates from our preliminary cryostructure of the CSN obtained from the isolated complex from human erythrocytes (Rockel et al., 2014). The sequence comparison is based on the comparison of amino acid sequences.

The LID and the CSN consist of six proteasome LID-CSN-initiation factor 3 (PCI) domain and 2 MPR1/PAD1 N-terminal (MPN) domain subunits (Figure 1) (Glickman et al., 1998; Hofmann and Bucher, 1998). The PCI domain proteins of the human LID are RPN3, RPN5, RPN6, RPN7, RPN9, and RPN12, whereas those of the CSN are CSN1-CSN4, CSN7A, and B, as well as CSN8A and B. The MPN domain proteins in the LID and CSN, namely, RPN11 and RPN8 or CSN5 and CSN6, respectively, form heterodimers (Forster et al., 2013), which are embedded into the “horseshoe” structure formed by the PCI domains (Figure 1). The MPN domain proteins are discussed in detail in the next section. Interestingly, so far, there are no paralog subunits in the LID as they were detected in human CSN: CSN7A and CSN7B, as well as CSN8A and CSN8B. They determine appropriate variants called CSN^CSN7A^, CSN^CSN7B^, CSN^CSN8A^, and CSN^CSN8B^, which occur simultaneously in most cells (Dubiel et al., 2023). The PCI domains are ordered in the LID as the RPN9/RPN5/RPN6/RPN7/RPN3/RPN12 ring (Figure 1) (Forster et al., 2013). A similar arrangement can be found in the CSN as CSN7/CSN4/CSN2/CSN1/CSN3/CSN8 (Lingaraju et al., 2014). In the LID and CSN, a helical bundle is formed by the C-termini of all subunits and lies over the PCI structure, the “horseshoe” (Figure 1) (Lingaraju et al., 2014). Under normal conditions, free subunit concentrations of the LID and CSN complexes are very small and have no additional effects on cells. Only under pathological conditions can individual subunits be over- or underexpressed and exert disease-causing influences.

In the LID, the N-terminal domain of RPN6 binds to the CP subunit α2 and holds the RP together (Forster et al., 2013). RPN5 can also interact with the CP depending on 26S proteasome conformation (Greene et al., 2019). Many aspects of deubiquitylation of polyubiquitylated substrates by Rpn11 remain unclear (Forster et al., 2013). The MPN domain of Rpn11 is located over the AAA-ATPase N ring of the BASE, suggesting that one major function of the LID is to hold RPN11 in place. RPN8 is inactive but might contribute additional ubiquitin-like-binding interfaces (Forster et al., 2013). Disrupting LID-BASE interactions by mutations leads to significant degradation defects of the 26S proteasome (Greene et al., 2019). LID formation, probably also the CSN assembly, is driven by the helical bundle (Estrin et al., 2013).

The CSN interacts with the CRLs to deneddylate cullins (CULs), but it is also associated with unneddylated CRLs (Dubiel et al., 2023). Although the best described interactions between CSN and CRL are defined in a recombinant complex of CSN^CSN7A^-CRL4A (do not react preferentially with each other) (Dubiel et al., 2023), the *in vitro* data can be taken as a first clue (Cavadini et al., 2016). The major contacts between CSN and CRL are formed with CSN2 and CUL4A C-terminal domain. CSN2 and CSN4 sandwich RBX1, and CSN1 contacts DDB1 (Cavadini et al., 2016). In the presence of CRL4A, conformational changes in CSN2, CSN4, and CSN7 affect the CSN5-CSN6 dimer and activate CSN5. Thomä and co-workers call it induced-fit activation of CSN (Cavadini et al., 2016). The active CSN5 deneddylates CULs, which inhibits the ubiquitylation activity by the CRLs. Now, the reassembly of CRLs is possible. Under these circumstances, tight binding of cullin-associated NEDD8-dissociated protein 1 (CAND1) to both the N- and C-terminal ends of CUL1 takes place. CAND1 expels the old substrate receptor (SR) and sequestered CUL1 in an inactive state. Renewed neddylation and, eventually, binding of a new SR yield an active CRL (Cope and Deshaies, 2003). A detailed model of the CAND1-mediated substrate receptor exchange was recently formulated (Shaaban et al., 2023). Neddylation of CUL in the CRL potently stimulates the transfer of ubiquitin from E2 to the substrate (Duda et al., 2008; Saha and Deshaies, 2008). Under the neddylation condition, CSN is associated with CRL (Dubiel et al., 2023). Interestingly, inhibition of deneddylation by CSN5i-3 (Schlierf et al., 2016) blocks ubiquitylation of the substrate p27 by CRL4A, although the neddylation is maximum (Wang et al., 2021; Dubiel et al., 2023). Thus, a constant sequence of neddylation and deneddylation seems to drive substrate ubiquitylation by the CSN-CRL complex.

## The MPN^+^/MPN domain protein module

The MPN^+^ domain protein, the catalytic active part of the MPN^+^/MPN module, possesses the sequence H-x-H-P-x [6]-S-x [2]-D, which coordinates an essential Zn^2+^ ion in its active site (Forster et al., 2013). The MPN domain protein seems to be inactive; it has no Zn^2+^ ion but might exhibit additional ubiquitin-like-binding sites (Clague et al., 2013). The two proteins form a heterodimer, which we call the MPN^+^/MPN module. Many protein complexes are equipped with MPN^+^/MPN modules, although their exact function is not yet clear. Most of the MPN^+^ domain proteases are activated in multi-subunit complexes by allosteric regulation via their specific substrates, which are sensed by other subunits of these complexes. Incorporation of MPN^+^ metalloproteases into multi-subunit complexes very likely helps discriminate between ubiquitin and ubiquitin-like proteins, bringing substrate specificity.

There are three paralog complexes, the LID, CSN, and the eukaryotic translation initiation factor 3 (eIF3), in which the module performs deubiquitylation (LID and eIF3) or deneddylation (CSN). Their MPN+/MPN modules are paralog proteins of LID, RPN11, and RPN8, of CSN, CSN5 and CSN6, as well as of eIF3, eIF3f, and eIF3h, respectively. LID, CSN, and eIF3 are also summarized as ZOMES complexes.

It is still unclear whether all MPN^+^ and MPN family proteins are present in large protein complexes and whether the inactive MPN domain protein performs a special function in these particles. Additional MPN^+^/MPN modules exist in the protein complexes BRCA1-A and BRISC. In both complexes, the MPN^+^ domain protein is BRCC36, which specifically cleaves ubiquitin Lys63 linkages. BRCC36 is activated in the BRCA1-A complex by the MPN domain protein ABRAXAS and in BRISC particles by the MPN domain protein ABRO1 (Rabl, 2020). Mutations on the MPN domain of ABRAXAS and ABRO1 lead to damage of deubiquitylation activity by BRCC36 (Pan et al., 2022). Although the MPN^+^/MPN module is very similar, the two complexes perform different tasks due to other subunits. Although BRCA1-A is recruited to DNA repair foci and edits ubiquitin signals on chromatin, the BRISC complex regulates the immune response, mitosis, and hematopoiesis (Rabl, 2020).

There are exceptions such as AMSH and AMSH-LP (AMSH-like protein), which seem to cleave Lys63-linked ubiquitin chains independent of MPN protein partners (Pan et al., 2022). AMSH and AMSH-LP have a similar structure. Their catalytic domains are completely conserved (Nakamura et al., 2006). AMSH mediates receptor endocytosis, which is accomplished through the recognition of specific ubiquitylation patterns, specifically multi-monoubiquitylation and Lys63-linked polyubiquitylation (Pan et al., 2022).

Unfortunately, too little is known about the MPN^+^/MPN modules. In particular, it is unclear what the role of the MPN component is.

Dysregulation of MPN^+^/MPN modules is implicated in several human diseases, highlighting the importance of MPN^+^/MPN module function. For example, tumorigenesis is caused by mutations of CSN5 and CSN6. Inflammatory diseases relate to overexpression of BRISC (Pan et al., 2022). Hence, therapeutic targeting of MPN^+^/MPN modules and developing of specific inhibitors give hope to fight various diseases.

## Is the COP9 signalosome a regulator of the 20S/26S proteasome?

In earlier experiments, we have shown that the CSN interacts with the 26S proteasome (Huang et al., 2005). Previously, evidence for the physical association of the CSN, the proteasome, and E3 ligases was provided in plant. A possible exchange of the LID and CSN was speculated (Peng et al., 2003). We isolated the human 26S proteasome and the human CSN and incubated the two complexes in different molar ratios together with ATP. We measure the decrease of ATP-dependent peptidase 26S proteasome activity by CSN. Subsequent immunoprecipitation revealed co-precipitation of the 26S proteasome and CSN. In particular, in a molar ratio of the 26S proteasome and the CSN of 1:20, a clear co-immunoprecipitation and inhibition of 26S proteasome peptidase activity was measured (Huang et al., 2005). Since subunits of the LID disappeared in the immunoprecipitation and subunits of the CSN became visible, we concluded an exchange of the LID by the CSN. In addition, we assume the existence of super complexes consisting of the 26S proteasome, the CSN, and selected E3s that carry out specific proteolysis (Huang et al., 2005). Unfortunately, a molar ratio of the LID to CSN of 1: 20 in cells is very rare, and the substitution of the LID by the CSN is unlikely. For example, Seaki and co-workers determined 140–200 nM in the cytoplasm and 830–980 nM in the nucleus of the total 26S proteasome concentration in yeast (Pack et al., 2014). The concentration of CSN was estimated in human cells to be 500 nM (Bennett et al., 2010). Under these conditions, only a very minor replacement of LID and CSN is conceivable. In addition, does the CSN perform deneddylation or deubiquitylation or both on the proteasome? Does it degrade specific proteins? Detailed research into the interaction of the proteasome with the CSN could provide important insights into new regulators of the proteasome. Unfortunately, the exchange of the CSN by the LID has not yet been investigated. In the future, it would be interesting to study the influence of LID on isolated CSN-CRL complexes with appropriate substrate ubiquitylation.
